# Intergenerational Commensality: A Critical Discussion on Non-Familial Age Groups Eating Together

**DOI:** 10.3390/ijerph18157905

**Published:** 2021-07-26

**Authors:** Simon Biggs, Irja Haapala

**Affiliations:** 1 School of Social and Political Sciences, The University of Melbourne, University Park, Parkville, VIC 3010, Australia; irja.haapala@unimelb.edu.au; 2 School of Applied Educational Science and Teacher Education, The University of Eastern Finland, 70100 Joensuu, Finland

**Keywords:** intergenerational, commensality, life-course, meals, generations, eating, non-familial, non-kin, family, food

## Abstract

Connecting intergenerational relationships and commensality has been a neglected area in research and conceptual development within both food and life-course studies. This has been especially true of relations beyond the family. Here, public and private settings are explored in order to examine the relationship between eating together and generationally intelligent empathy. This is to help the discovery of spaces where different generations can interact positively around food and mealtimes. Contemporary social and public health challenges include: to adapt to increased longevity and to build solidarity between generations; to repair the relations between generations arising from institutional segregation; and to increase experiences of generational connection and social inclusion. As age-based cohorts are led to see themselves as separate from each other, we must find ways of building and negotiating new complementary roles for different parts of the life-course. Commensality, eating together at the same table provides an important cultural location and opportunity around which complementary understandings between generations may be built. A new framework is proposed to help identify and critically examine the variables underpinning non-familial intergenerational commensal spaces.

## 1. Introduction

Population aging, the relationship between generations, and healthy eating are important public health issues for the 21st century [1]. Generations have a problem considering that spaces for shared positive interaction are limited [2,3,4]. Commensality often implies intergenerational relationships [5] but in research this is rarely made explicit except in family contexts. The question of how to identify opportunities for different generations to eat together as an aspect of promoting public health through commensality is a much-needed but under-researched area in both food studies and social gerontology. Commensality is, at the root, about interpersonal interaction around food and drink that are consumed together with other people [5]. It goes beyond decontextualized food choices and varies depending on an individual’s place in the life-course, chronological age, physiological status, and life priorities at a given time. While food is primarily consumed to satisfy our physiological needs, eating alone or with others in many ways reflects our psychosocial history and relationship to food both in the public and private sphere. In this context, people of multiple generations eating together has the potential to promote shared healthy food habits and increase social inclusion. This paper draws upon two parallel but rarely connected disciplines: food studies and studies of the adult life-course to increase understanding of cognate social issues.

In this paper, we will critically examine the treatment of intergenerational relations in existing literature on commensality and suggest a framework to help understand generations in the context of shared meal-taking. The framework helps to identify and critically examine factors underpinning commensality in intergenerational settings in both private and public spheres. The private domain will be extended beyond single household families to include multiple sites of intergenerational interaction around meals.

The proposed framework focuses on non-familial intergenerational interaction to increase its relevance to public health in the context of wider social attitudes such as those pertaining to generational difference and connection. Together with commensality, intergenerationality influences people’s decision-making about food purchasing, preparation, and consumption, and our principal emphasis here will be on the two latter points with a focus on identifying spatial connections in life experience and life priorities between generations. We argue that the promotion of different age groups eating together in a shared location is a key component of healthy living through the sharing of current and future life projects, and that the practice is an important part of building an interconnected society.

## 2. Framework for Identifying and Examining Variables Underpinning Non-Familial Commensal Intergenerational Spaces

One of the first tasks in examining non-familial intergenerational commensality, given the tacit social experience and paucity of research on this topic, would be to develop tools to map the sites and contexts where intergenerational commensality may take place. As presented in Table 1, this can involve asking the question of who, where, what, when, and how intergenerational commensality occurs.

**Figure 1:**
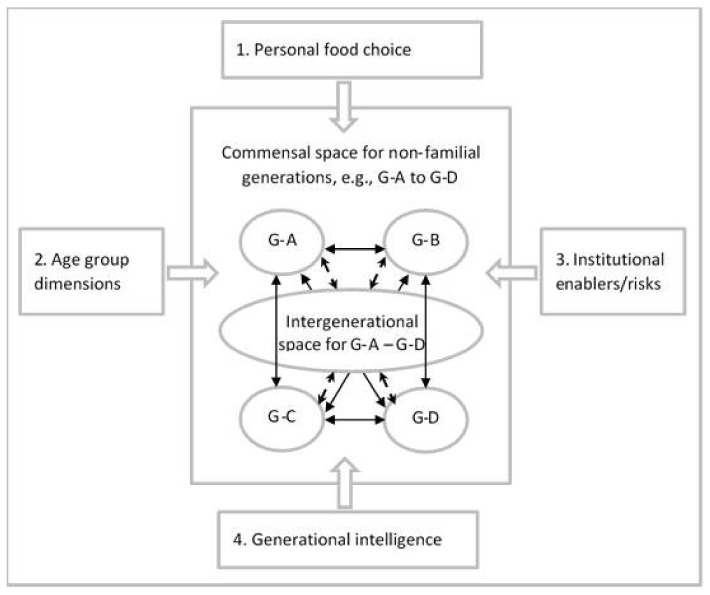
Identifying and examining variables underpinning non-familial commensal intergenerational spaces.

Within the identified intergenerational commensal settings, the first step would be to examine the possible evidence-based variables underpinning the activity. In our proposed framework, presented in Figure 1, these variables relate to factors affecting (1) personal food choice (based on work by Sobal et al. [6]), (2) age group dimensions, life-course and family position in addition to cohort experience (based on work by Elder et al. [7]), institutional enablers/risks (based on work by Haines [8,9]), and the degree to which participants act with intergenerational awareness (based on work by Biggs et al. [10,11]). For more detail on these variables, please see Table 2 and the critical discussion that follows.

1. G-A–G-D refer to distinctive generational groupings based on age group dimensions (see Table 2).
2. Intergenerational space refers to shared commensal activity between age groups.
3. Solid line arrows indicate that two age groups interact.
4. Dash line arrows indicate that several age groups interact.

The framework arises from our empirical and conceptual work in food and life-course studies. Long-term influences on food habits and nutritional well-being in the adult life-course were studied in a 21-year longitudinal study in Finland among 103 women from the original FINMONICA cohort of 299 women who were 50–60 years of age at the start of the study [12], accompanied by in-depth interviews of ten women after the 32-year point for a follow-up conducted by the second author. This indicated a strong influence from personal food system and values including relationship management, delineated by life-course related variables (age group dimensions) [13,14]. In two of our studies that engaged adolescents with older adults in rural Finland and international community services in the UK, we used the generational intelligence model to build empathy and strengthen intergenerational understanding regarding home-care tasks [15]. By engaging participants in the generational intelligence process of considering theirs and the others’ generational position and priorities ahead of the interaction, and in the post-intervention interviews, we were also able to better assess the outcomes. In a further study on nurse education in community settings in Hong Kong, in which the training and the design of pre-post questionnaires were based on the generational intelligence model, the outcomes demonstrated an increase in empathic understanding between health professionals and older care recipients [16]. Our research on age-related encounters in public and private urban settings examined naturally occurring generational attitudes between disadvantaged youth and older adults in Melbourne, Australia. Interviews highlighted both a lack of and a genuine wish for more opportunities for intergenerational encounters in public settings [3]. Further research on the use of urban space by older people and children [4] in addition to the study of managing organizational risk in the regulation of dementia care [17,18] also contributed to the final framework. An analysis of social policy in relation to intergenerational relations can be found in Biggs [10]. The proposed framework includes the variables we have found of interest as influencing intergenerational encounters including mealtimes, now extended to non-familial commensal meal-taking.

Our work in this field links to the American literature on intergenerational projects, collated in a recent publication by Kaplan et al. [2]. Here, “Intergenerational Contact Zones to promote place-based strategies for social inclusion and belonging” are studied, connecting to our focus on the specifically non-familial relations in commensal situations and the related psychosocial aspects.

## 3. Personal Food Choice, Commensality, and Generations in Existing Research Literature

Personal food choice guides what we eat and commensality provides us the social setting and company for the meal. A commensal meal has been described as “a structured event that is a social occasion which is organized according to rules prescribing time, place and a sequence of actions” [19] and somewhat more colloquially as “the foundation of socialization, in the twofold meaning of the word: the place to learn the rules of living together and the place for social interaction, for sharing and for friendly exchange” [20].

In an earlier article touching on commensality, Sobal et al. [21] spoke of personal food choice in terms of chains, cycles, webs, and context (or ecology), and went on to develop a systems model that includes social environments [6]. Variables important to people’s personal food choice included “personal food systems” (making value judgements on balancing food choice values including taste, convenience, cost, health, and relationship management); external and internal “influences” including ideals, resources, social factors, and contexts; and personal factors (age, health status, preferences, cravings, etc.). These were shown to be further guided by people’s life-course, generation, and cohort experiences including experiences from childhood and years accompanied by war. The authors argued that each influence would vary depending on an individual’s experience of the aging process and their life-course events [6].

Sobal et al. [6] drew on the work of social gerontologist Glenn Elder [22,23] to explore a life-course dimension to food choice. Attention was provided to trajectories across the life-course; transitions provoked by particular life events that can create turning points in lifestyle and food choice; timing of an event in a particular life-course such as pregnancy; and context that refers to historical cohorts. Micro-contexts were also identified including “families, school, work, community, etc.”. However, these contexts were internally undifferentiated and subdivisions or cross-cutting categories were rarely examined. This is unfortunate considering the current study as generational difference was not mentioned apart from the fact that many of the social institutions that were identified were generationally specific. It is strange, though, as one of the most significant discoveries of Elder’s original cohort studies was on the ways that the timing of an historical event such as an economic depression has different impacts on individuals depending upon age-group, family position, gender, and cohort experience.

Generations are tacitly central to studies on commensality through its emphasis on family meals [24] and the life-course [6]. Studies of age group and generational commensality, however, are rare and where they are mentioned, they are often not expanded upon. Families arise within this discourse as a site of research but also as a model structuring non-familial generational relations. Explicit study of generational commensality is almost unknown. Germov and Williams’ [25] handbook *The Sociology of Food and Nutrition,* for example, mentions neither generation nor cohort in its index, though it does refer to family. Mäkelä [26] has referred to the notion of a “proper meal” being closely connected to family life and describes non-familial commensality as “a kind of togetherness, a lovers’ dinner, a teenagers’ hamburger meal at a fast-food place, a lunch with colleagues” (p. 41). Furthermore, commensality in the workplace [27,28] and at school [29,30] has been studied but without explicit reference to the respective ages of the participants involved. In each case, commensality has been found to increase nutritional intake and sense of well-being. What is striking is that each of these examples is as generational as family mealtimes but their intergenerationality is left unexplored.

The value and possible risk of commensality for intergenerational connection can be seen in Niva and Mäkelä’s [31] suggestion that “while not all meals are characterized by conviviality and companionship, they continue to serve as a significant area of human sociability and togetherness”. Family meals, often normatively but sometimes mistakenly thought of as haven for convivial transfer of knowledge from generation to generation [32], in the current context of demographic shift and migration patterns, may become less common than non-familial meals. Still, European family meal-taking has been reported to be both resilient and by no means in decline [24,33], while Holm et al. [33] found that contemporary non-familial meal-taking comprised approximately 56% of all contexts including eating alone or with colleagues, friends, and others. Much of the recent research in meal studies has focused upon eating alone [34], commensality with computers [35], and virtual meal-taking [36]. This interest in the possibilities of indirect non-familial commensality would seem to be pushing a concept that combines eating together to areas where commensality is thin and where the boundaries between private and public meal-taking have become blurred. Thus, where might research tell us that non-familial intergenerational commensality takes place?

Much of the study of intergenerational sharing apart from families has been at the beginning and later stages of the life-course and involved institutions including projects for kindergarten and elderly care facilities [37,38]. Oropilla’s [39] review of older people and children’s interaction concluded that the places for intergenerational interaction are limited and that children’s voices are rarely heard. Intergenerational relations are, however, a priority referenced in the United Nations’ [40] work towards achieving their 2030 Agenda and 17 Sustainable Development Goals (SDGs). Oropilla reported that “Of the 17 SDGs, five are closely linked to intergenerational research: SDG 1 No Poverty, SDG 2 Zero Hunger, SDG 3 Good Health and Well-Being, SDG 4 Quality Education and SDG 16 Peace, Justice and Strong Institutions”, often via family policies to enable collaborative efforts between generations [39]. The fact that each of these goals are connected to food and meal-making is also clear. Nevertheless, these have been elaborated almost exclusively in terms of family policy.

A much-needed correction to this approach would be a conceptual framework for finding sense in the intergenerational dimension of commensality, clarifying it explicitly, and reaching out beyond the boundaries of family, thus looking across the different parts of the life-course and into more public arenas.

## 4. Age Group Dimension: Life-Course, Cohort and Family Position

Generational work involves at least three understandings of personal and social experience: those of life-course, cohort, and family position. Life-course refers to the priorities and life tasks most salient at any one point in a human lifespan; cohort, refers to the social experience of living through particular historical periods; and family position refers to being a child, parent, grandparent, or great-grandparent [11]. Each of these has an influence on personal and social experience.

Using the Oakland Growth Study, begun in 1920, Elder [22,23] was able to track changes in people’s lives as they encountered significant social change. He found that the timing of major social events interacted significantly with an individual’s life-course position. Factors such as birth order, age, and gender affected future life chances depending upon when the events took place. Children were affected by the degree of responsibility they were expected to take within their families and how their same-gender parent was affected. A network of shared relationships meant that “each generation is bound to the fateful decisions and events in the others’ life course” [22] (p. 40). Longitudinal studies of life chances following the Great Depression, the Second World War, and the economic downturn in the 1980s in the American Midwest illustrated how individual lifetimes, family time, and historical time interconnected. Whether one was a younger or older war recruit determined whether events occurred advantageously or at “untimely points” for future prospects, and whether one was at the crest of a wave or always challenged by younger cohorts determined long-term disadvantage.

In a scenario reminiscent of recent recessional trends and the pandemic, for example, individuals could receive college degrees and have no place to move forward in a stagnant economy. The work of Elder [7] and Attias-Donfut and Wolff [41] in France demonstrated that the protective role of the family can be uneven, equivocal, and can only be understood in close relationship to an individual’s life stage and cohort history. Responding to historical events and the risks they represent is itself shaped by the role of family members in passing the experience of social events on to younger generations with oral histories and shared experiences acting as an important filter for official versions of events [7,41].

Arguably, renewed risks associated with recession, increasing inequality, climate crises, and pandemics has highlighted the complex and ambivalent nature of intergenerational relations in the context of changing commensal practices and attitudes to public health. Multiple factors shaping how generations connect have led to a re-focusing on methodological and conceptual innovation that highlights the importance of transitions and continuities in shaping age-based relationships [42]. A need to focus on the emotional value of such relations and the discovery of sites where positive interaction can take place has been added to this school of thought [2,10].

## 5. Institutional Enablers/Risks, Ageism, and Age Segregation in Generational Relations

Fiona Haines [8,9] has argued that in order to understand and ultimately change our responses to situations in which risk should be managed, three areas need to be considered. First, there is technical expertise: this includes the scientific data available on which to make a choice and the capacity to implement a course of action. Second are sociocultural expectations: whether there is good fit or not between the proposed actions and the environment into which they would need to be embedded. Third, political will is necessary to balance markets and legislation in favor of planned activities and the direction of policy. Having knowledge and expertise in the advantages of phenomena such as eating together or connecting different age groups is not going to achieve change on its own. It has to achieve an acceptable fit with pre-existing attitudes and the processes that change them, in addition to a means of generating a momentum to redirect policy and resources.

Improving intergenerational relations has been complicated by the fact that age-based differences have historically been associated with imbalances of power [43,44,45]. Furthermore, understandings of ageism, defined as the existence of prejudice or discrimination based on age, varies depending upon the disciplinary discourse encountered. In youth studies, ageism is predominantly interpreted as attitudes that disadvantage younger adults [46]. In gerontology, ageism is presented as a phenomenon affecting older adults [47]. In an attempt to make ageism age-neutral, Biggs [48] (p. 96) has suggested it is: “The colonization of the goals, aims, priorities and agendas of one age-group by another… This may be consciously done for reasons of political and economic expediency, or unknowingly as if these priorities are simply common sense”, while Phillips et al. [49] have associated it with the psycho-social process of “othering”.

A key factor that has contributed to a lack of understanding and commonality between generations has been the institutional separation of age groups: children are segregated by age within the education system, older people have often been separated into specialist age-care settings, and mid-lifers have been placed in the age-neutral or age-ambiguous spaces of work and non-work [50]. This reduces contact between generational groups, thus reducing the possibilities for countering public stereotypes and enhancing interpersonal empathy [3,10].

Coverage of age differences in the public sphere has most often been negative [44,51] and, with the inception of the Global Financial Crisis from 2008, has taken a political turn that located social inequalities within a generational framework [52,53,54,55,56]. In other words, generational difference has been used as an interpretive tool in an attack on issues seen to affect age groups unevenly such as age-specific benefits including pensions, payments for university education, housing availability, the casualization of work, and eroded public sector services.

These changes in attitudes between generations and absences of positive intergenerational contexts have led to calls for new forms of positive generational activities to be found [57,58,59,60] in order to actualize positive technical expertise, shift cultural expectations, and encourage political will. A combination of institutional segregation in non-familial settings and the ebb-and-flow of generational politics emphasizes a need for both increased generational empathy and the discovery of events and locations where different age groups can meet and enjoy each other’s company as an antidote to negative messages and structures in the public sphere. Commensal meal-taking has the potential to be one such site for building positive and lasting intergenerational relationships particularly if enabled by institutional support. Positive forms of commensality can counteract ageism and institutional age segregation. Othering could be construed as a positive rather than a negative experience through recognizing the generationally specific contributions each “brings to the table” and psychological distance countered by personal sharing. Recognizing that others have age-specific priorities would also be an important contributing factor. Positive othering has been described as when: “The other person is approached as someone with her or his own characteristics, projects and idioms. Positive othering constitutes a willingness to consider distinctiveness based on adult age, even if these do not correspond with the dominant group’s interests and world view” [10] (p. 23).

There is some evidence that the process of positive othering may not occur naturally. Dow et al. [3] have found that positive intergenerational relations occurred much more easily in private rather than in public spaces and that the public domain required forms of activation such as those described by Kaplan et al. [2]. Work by Haapala et al. [15] on rural community services has indicated that both older customers and younger assistants might need education on each other’s life-course priorities for the improved delivery of home-based support. Similarly, Au et al. [16] have shown how supporting young carers through education on how to consider the perspective of the other (care recipient) can be beneficial to both parties. From the perspective of generational relations, commensal interaction around meals may provide a linking narrative across public and private spheres with the possibility of a space that allows for sharing, complementarity, and mutual learning.

The study of groups, beginning with the seminal work of Sherif and Sherif [61], has indicated that contact in itself is not enough for positive commensal behavior to take place. Sherif and Sherif found after artificially provoked group rivalry between teenagers at a summer camp that simply meeting up over meals could inflate existing rivalries. Shared mealtimes became excuses to act out rivalry with sandwiches as projectiles rather than generating harmony. Finding a superordinate goal that both groups had to complete together to achieve a shared result allowed harmonious relations to re-emerge before the parents retrieved their offspring [61]. Bearing this in mind, a positive commensal space would need to include the meeting of shared needs and mutually rewarding consequences.

In generational terms, achieving a positive commensal space would mean addressing multiple forms of external influence as identified in our proposed framework (Figure 1) where successful intergenerational commensality would provide an opportunity to rise above age stereotyping and rivalry to more powerful forms of cooperation and enjoyment through shared meal preparation and consumption. The aim would be to guide participants to act with a background awareness of generational particularities and complementary skills but nevertheless act as if they were not of principal concern. Participants would be invited to an event to enjoy good food and company, for example, instead of to go to a “generational learning event” accompanied by a light meal. Discussion topics over the meal arise as chosen by the participants. Work by Mintz [62] and Fischler [63] has shown that commensality can produce some of these positive outcomes, arguing that it enhances group identity. However, the value of creating an exclusive in-group has been questioned [64] and the need to recognize diversity has been identified [29] both for food choice and social identity.

## 6. Generational Intelligence and Building Empathy in Intergenerational Commensality

In considering Elder’s and Sherif and Sherif’s observations [22,23,61], in addition to trends in the public discourse, there comes a suggestion that increased empathic understanding or putting oneself in the shoes of the generational other would be an important contributor to achieving a shared positive commensal activity. One way of addressing degrees of empathic understanding between people of different generations and a search for appropriate settings for interaction can be found through the study of generational intelligence [11].

Generational intelligence denotes “an ability to reflect and act in a manner, which draws on one’s understanding of one’s own and other’s life-course, family, social history, placed within a contemporary social climate” [65]. The idea is further elaborated regarding the different degrees that social actors behave reflexively with respect to their sense of identity and social situations such as meal-taking. Such a reflexive awareness of generations creates room for maneuver. It creates a distance between assuming one’s own attitudes and values are universally shared and being able to step back in order to take a sustainable value position. It turns fixed positions into options.

Biggs and Lowenstein [11] have observed that each life phase has a distinctive set of priorities due to its position in an expanded lifespan. Once this is recognized, it emphasizes that generations differ and any one single perspective cannot be assumed to be universally valid. Thus, generational positions will require negotiation between each other. The degree to which people become aware of this and their willingness to positively encounter the age-other indicates their degree of generational intelligence. Empirical work using generational intelligence has been undertaken by Haapala et al. [15], Au et al. [16] and Dow et al. [3] to examine intergenerational relations in social work, education, and community settings. According to this model, any person or set of social arrangements can contain high or low degrees of generational intelligence. Low generational intelligence is evidenced by a lack of capacity to recognize others beyond ones’ own needs and perspective, whereas high generational intelligence is evidenced by an ability to empathize with an alternative life-position and build complementary relations between oneself and others that can stand the test of time.

In other words, having high generational intelligence allows the thoughts, feelings, and values associated with a commensal space, the degree to which people are aware of it, how they react to it, and the effect it has on the sense of who they are and how they behave towards others to form a basis for what might be called positive generational commensality. It forms a bridge between different life priorities and shared food-related activities.

Rather than thinking in terms of cohorts, families, or individual life positions as separate sources of generational identity, it is argued that people experience them “all in one go”. This implies both a holistic experience and one that is often tacit rather than consciously thought through. Thus, in terms of generational categories, an individual in life-course terms may be in midlife, in family terms be part of a sandwiched generation, and belong to generation “x” as a cultural category. As a phenomenological space, she or he may change from focusing on times in childhood to looking forward to the amount of time they think they might have left, wondering how competing family demands will allow them to use this time to best advantage and identifying with younger rather than older generations, striving for self-actualization. Their awareness of self and others is generationally inflected and is an amalgam of influences that have yet to be designated or understood but nevertheless affect intergenerational behavior even if it is not always actively thought about. Thus, for such a person a commensal meal might draw on their assumptions about advantageous food purchasing and healthy and enjoyable food habits based on their being in their middle years, accommodating or assimilating the food preferences of other groups around the commensal table, particularly younger and older generations, in addition to the historical experiences that have led to them preferring a particular cuisine or location.

The process of generational intelligence includes four elements as presented in our framework (Figure 1 and Table 2): self-awareness of age and life-course position; empathic understanding of others’ generational priorities; value judgement and positions for interaction; and negotiation for mutually beneficial and agreed-upon solutions and goals [65].

The first of these would be for social actors to locate themselves within a distinguishable generational group based on an amalgam of life-course, cohort, and family position. This would help to clarify the specific existential tasks associated with one’s life circumstances.

Once one has become aware of one’s own generation, the question of how to relate to those who are different from you on this dimension arises. Empathic understanding can be built by increasing explicit awareness of generational distinctiveness and a need to balance difference and similarity of interest; in turn, this avoids the trap of thinking that all age groups hold essentially the same priorities and needs. It recognizes the age-other as more than an extension of one’s own priorities and reflects a relative ability to put oneself in the position of other generations.

The third element includes making a value judgement in terms of one’s position on the quality of this relationship in terms of conflict and cooperation. A non-conflictual resolution would require holding potentially contradictory elements in mind without succumbing to oppositional thinking. In terms of increased generational intelligence, this process would include the development of complementary relationships within particular settings.

A final process would involve intergenerational negotiation. Such negotiation would need to be mindful of the opportunities for recognizing distinctiveness while simultaneously discovering the enjoyment of shared activity. It would begin from the perspective that the projects of different generational groups are equally valid, yet accommodation towards sustainable solutions requires compromise.

In this paper, we argue that intergenerational commensality can provide a vital opportunity to increase positive and enduring relations between non-familial generations in public and private spheres. In so doing, we are not arguing for a return to traditional family meal-taking, although it might take family-like forms. Rather, we would understand commensality as a powerful mechanism for prefiguring the development of positive generational relations in both the here-and-now and in consideration of future relationships, apart from in the public imagination. To create a lasting positive relationship between potentially conflicting generational groups, mixed feelings must be acknowledged and negotiated. According to the process presented here, this would rely upon increased personal generational awareness, recognizing complementarity, and a willingness to negotiate a shared solution as a part of the practice of commensality.

Positive solutions within intergenerational commensality can be based on recognizing the complementary nature of what each participant brings to the table. This can be based on generational priorities, perspectives, or skill sets. There can be many reasons why both positive and negative generational relations take place and are maintained, with lasting positive relations based on containing such ambivalence. They must be construed as in some way positive in terms of their purpose, value, and outcome. They require negotiated solutions so that each generational perspective is recognized and respected, while being accommodated or assimilated into a mutually acceptable set of superordinate goals, for example, around the production, distribution, purchasing, preparation, or consumption of food. There must be agreed-upon processes and mechanisms for resolving contested expectations. Similarly, commensality can be evaluated depending upon the degree that it enables a space where multiple aspects of generational identity can be recognized and valued.

In general, the research into commensality has concluded that eating together with others produces beneficial health effects and bonding, suggesting that it could also contribute to enhanced understanding between generations especially when these exist beyond the confines of familial relationships. There are many research questions that arise from this critical discussion that would benefit from the collaboration between food and adult life-course studies. These might include: What is the relationship between naturally occurring and organized intergenerational commensal situations? To what degree do groups need to be facilitated and by whom? What is the role of a facilitator, convener, or enabler of future intergenerational non-familial commensality in orchestrating positive encounters and lasting relationships? What are the contexts that are most conducive to generational understanding? Which elements of the meal are most conducive to both commensality and intergenerational connection, production, purchasing, preparation, consumption, and so on? Both commensality and intergenerational connection claim to have beneficial health effects; thus, do the consequences of combining them include additive consequences, summative consequences, or what else? Which mix of age groups is most effective for intergenerational commensality? What are the short, medium, and long-term consequences in terms of relationships between generations and health?

## 7. Conclusions

We have proposed a framework that draws on the two fields of food studies and adult life-course studies, aimed at researching into non-familial intergenerational commensality. This framework can be used as a means of identifying and critically examining variables underpinning intergenerational commensality that are currently conceptually and empirically underdeveloped. We suggest that spaces for non-familial intergenerational commensality require further research in the public and private spheres in terms of what occurs there and how can they operate to address public health issues associated with aging societies, food and healthy living.

Taken together, intergenerational non-familial commensality is desirable in so far as it provides a potentially healthy and positive shared space that increases generational connection and social inclusion. It supplies a common superordinate goal in the form of successful meal-taking, providing a natural space for discussing public health issues and building practical solidarity between generational groups in everyday situations. The framework presented here, based on the authors’ and others’ empirical and conceptual work, holds the potential of allowing the contribution of two largely separate disciplines of food and life-course studies to be combined to aid future researchers in public health and related fields.

## Figures and Tables

**Table 1:** Mapping the context of non-familial commensal intergenerational spaces.

The context: What are the features of the commensal site? *Who*: Who is present in the commensality space? Which generational groups are present? What are their life-course priorities and perspectives? *Where*: Where is the commensal encounter taking place? What are the salient generational features of that space (phenomenological salience)? *What*: What happens during the commensal event? What is its stated/principal purpose and consequence? What are the priorities of the different members regarding the event? *When*: At what time does the event take place? How long is it? How often does it take place? *How*: What must happen for the commensal event to take place? How are roles distributed? How are expectations communicated?

**Table 2:** Variables underpinning non-familial commensal intergenerational spaces.

Personal food choice External and internal influences: ideals, personal factors, social framework, food contexts, and resources Personal food system: values, sensory preferences, convenience, cost, quality, healthiness, relationship management, and so on Age group dimensions Life-course position (childhood, adolescence, young adulthood, early and late mid-life, and old age) Family position (child, parent, grandparent, great grandparent, etc.) Cohort experience (historical events, culture, social climate, etc.) Institutional enablers/risks Technical expertise, cultural expectations, and political will Generational intelligence Self-awareness of age and life-course position Empathic understanding of the other’s generational priorities Value judgement and positions for interaction Negotiation for mutually beneficial and agreed-upon solutions and goals
